# Spatial analysis of factors associated with HIV infection in Malawi: indicators for effective prevention

**DOI:** 10.1186/s12889-020-09278-0

**Published:** 2020-07-25

**Authors:** Jerry John Nutor, Henry Ofori Duah, Pascal Agbadi, Precious Adade Duodu, Kaboni W. Gondwe

**Affiliations:** 1 Department of Family Health Care Nursing, School of Nursing, University of California, San Francisco, USA; 2 Research Department, FOCOS Orthopaedic Hospital, Accra, Ghana; 3 Department of Nursing, Faculty of Allied Health Sciences, College of Health Sciences, Kwame Nkrumah University of Science and Technology, Kumasi, Ghana; 4 East Surrey Hospital, Canada Avenue, Redhill, Surrey England, UK; 5 College of Nursing, University of Wisconsin-Milwaukee, Milwaukee, WI USA; 6 Center for Advancing Population Science, Medical College of Wisconsin, Milwaukee, WI USA

**Keywords:** Pre-exposure prophylaxis, Risk behavior, Rural, Gender, HIV

## Abstract

**Background:**

The objective of this study was to model the predictors of HIV prevalence in Malawi through a complex sample logistic regression and spatial mapping approach using the national Demographic and Health Survey datasets.

**Methods:**

We conducted a secondary data analysis using the 2015–2016 Malawi Demographic and Health Survey and AIDS Indicator Survey. The analysis was performed in three stages while incorporating population survey sampling weights to: i) interpolate HIV data, ii) identify the spatial clusters with the high prevalence of HIV infection, and iii) perform a multivariate complex sample logistic regression.

**Results:**

In all, 14,779 participants were included in the analysis with an overall HIV prevalence of 9% (7.0% in males and 10.8% in females). The highest prevalence was found in the southern region of Malawi (13.2%), and the spatial interpolation revealed that the HIV epidemic is worse at the south-eastern part of Malawi. The districts in the high HIV prevalent zone of Malawi are Thyolo, Zomba, Mulanje, Phalombe and Blantyre. In central and northern region, the district HIV prevalence map identified Lilongwe in the central region and Karonga in the northern region as districts that equally deserve attention. People residing in urban areas had a 2.2 times greater risk of being HIV-positive compared to their counterparts in the rural areas (AOR = 2.16; 95%CI = 1.57–2.97). Other independent predictors of HIV prevalence were gender, age, marital status, number of lifetime sexual partners, extramarital partners, the region of residence, condom use, history of STI in the last 12 months, and household wealth index. Disaggregated analysis showed in-depth sociodemographic regional variations in HIV prevalence.

**Conclusion:**

These findings identify high-risk populations and regions to be targeted for Pre-Exposure Prophylaxis (PrEP) campaigns, HIV testing, treatment and education to decrease incidence, morbidity, and mortality related to HIV infection in Malawi.

## Background

Human Immunodeficiency Virus (HIV) and Acquired Immune Deficiency Syndrome (AIDS) continue to be a global health concern. According to the World Health Organization (WHO), out of the 36.9 million people living with HIV in 2017, more than half (19.6 million) live in sub-Saharan Africa [1]. The disease burden in sub-Saharan Africa has undermined the already slow pace of development in that region, reduced life expectancy, increased the number of orphans, and contributed to the destruction of families and community structures [2–4]. Eastern and southern Africa are some of the regions badly affected by HIV [5]. According to the Joint United Nations Program on HIV/AIDS, the HIV prevalence among adults in eastern and southern Africa was approximately 6% in 2018, with females disproportionately affected than males [6]. Malawi, a low-income country in southeast Africa, has one of the highest HIV prevalence rates among adults—estimated at 9%—with about 38,000 people newly diagnosed with HIV in 2018 [5].

Evidence has shown that socioeconomic and demographic factors are important predictors of HIV transmission, which necessitates interprofessional collaborations between basic and social scientists, including geographers in HIV and AIDS, in resource-limited countries. There has been considerable research to investigate the geographical variation of the HIV epidemic in sub-Saharan Africa [7–11]. After almost four decades of research, we have comprehensive knowledge of the factors associated with HIV infection in sub-Saharan Africa [12–14]. It is now imperative to investigate their geographical-level intensity and contributions to HIV to identify key populations. An important technique is to use a less costly spatial mapping approach to estimate the highly heterogeneous HIV prevalence and its drivers. By this method, we can critically examine spatial heterogeneity, identify the factors driving HIV spread in hotspots of the disease, and eliminate the masking of intra-regional differences.

Some studies in Africa have established that varied HIV risk factors associated with adult population may enable researchers to explain inconsistencies in HIV prevalence [15, 16]. For example, a study conducted in Uganda with the 2011 Uganda Demographic and Health Survey (DHS) and AIDS Indicator Survey (AIS), using spatial clusters and geographical variation of HIV showed several high and low rate geographical clusters of HIV infection—one significant cluster being those who used condoms less frequently and those who were uncircumcised [16]. In KwaZulu Natal Province, South Africa, spatial variations of HIV infections among women were investigated using geo-additive models [17]. These models identified significant geographically-focused patterns that would not have been accounted for by standard regression procedures [18]. The use of spatial cluster techniques to identify clusters of HIV prevalence enhanced the understanding of the determinants of HIV infection and geographic patterns, leading to improved resource allocation in Zimbabwe [10].

In Malawi, there is limited regional and spatial research using geographical and disaggregated analyses to better understand the spatial epidemiology of HIV/AIDS. Understanding this may help to guide health officials in formulating appropriate interventions to reduce new infections, and better allocate resources to support those who are living with the virus. Therefore, this study aimed to model the predictors of HIV prevalence in Malawi through a multivariate complex sample logistic regression and disaggregated analysis, and spatial mapping approach using the DHS dataset.

## Methods

### Study area

Malawi is a country located in southern Africa. Its geographical coordinates are 13°30′ South latitude and 34° East longitude [19]. The total land area is estimated to be 118,484km^2^. As a landlocked country, it is boarded by Zambia to the west, Tanzania to the north and north-east, and surrounded by Mozambique to the east, south and southwest [19]. Agriculture is the predominant economic activity accounting for a third of its GDP and 80% of its foreign revenue [19]. Agricultural activities are mainly dependent on rainfall. The staple crop is corn. The population size based on the latest 2018 population and housing census was 17,563,749 with an intercensal growth rate of 2.9% with 84% of the population residing in rural areas [20]. The country has three main administrative regions (Northern, Central and Southern) with districts at the sub-regional level [20]. The Northern Region comprises of districts such as Chitipa, Karonga, Nkhata Bay, Rumphi, Mzimba (that covers Mzuzu City), and Likoma. The central region has the following districts: Kasungu, Nkhotakota, Ntchisi, Dowa, Salima, Lilongwe, Mchinji, Dedza, Ntcheu, and Lilongwe. The southern region consists of the following districts: Mangochi, Machinga, Zomba, Chiradzulu, Blantyre, Mwanza, Thyolo, Mulanje, Phalombe, Chikwawa, Nsanje, Balaka, Neno, Zomba, and Blantyre. The four major city centres are located at Lilongwe, Blantyre, Mzimba (Mzuzu city) and Zomba and they constitute 5.6, 4.6, 1.3 and 0.6% of the total population of Malawi, respectively [20].

### Study data

This paper is a secondary analysis of an existing data set using the 2015–2016 Malawi Demographic and Health Survey (MDHS) and AIDS Indicator Survey (AIS). The DHS is a multi-round cross-country survey that is conducted to assess the general health of the population with emphasis on maternal health, child health and population health indicators of global health importance, such as HIV prevalence. Data collection was done by the National Statistical Office of Malawi in partnership with the Ministry of Health and the Community Health Services Unit of Malawi. The census frame included in the 2015–2016 MDHS (hereafter referred to as 2016 MDHS) is the total list of standard enumeration areas (clusters) demarcated for the 2008 Malawi Population and Housing Census. The 2016 MDHS adopted a multistage sampling involving the selection of clusters in the first stage and subsequent selection of households in the second stage. Cluster selection was also stratified by place of residence (i.e. rural/urban) and districts. In all, 850 clusters were selected in the first stage. This comprised of 173 and 677 from urban and rural areas, respectively. The probability of cluster selection was proportional to the cluster size and independent at each sampling stratum. A total of 26,361 households were selected using systematic sampling during the second stage of the sampling process.

### Measurements

The dependent variable in these analyses is HIV/AIDS status, which was measured for each participant included in the survey and was binary (negative/positive). HIV diagnostic testing was conducted using two rapid tests on whole blood sourced from either a finger-prick or venipuncture. The current analysis includes the following independent variables: socio-demographic, biological and behavioral factors. Socio-demographic variables were age, gender, place of residence, education level, religion, marital status and region. Behavioral factors included the use of condom for most recent sex, age at first sex, the total number of lifetime sexual partners and the number of extramarital sexual partners. Biological factors included the presence of a sexually transmitted infection (STI) or its symptom in the past 12 months. Socioeconomic status was assessed using the household wealth index per DHS criteria. Household wealth index were calculated and reported in the DHS data. This was estimated using household characteristics (source of drinking water, type of toilet, sharing of toilet facilities, main household material for roof, walls and floors floor, and type of cooking fuel amongst others household characteristics) and household assets (ownership of television, radio, vehicle, bicycles, motorcycles, watch, agricultural land, farm animals/livestock, and etc.). DHS used a factor analysis to assign weights to each asset in each household and aggregate score were calculated from the assigned weights. Households were ranked according to the cumulative scores from the household assets. The percentage distribution of the aggregate wealth score was calculated and values that corresponded to the four cut point values of the quintiles were determined. Households wealth was categorized using the following distinct cut points: less than or equal to the 20th percentile; greater 20% but less than or equal to 40th %; greater than the 40th % and less than or equal to the 60th % score; greater than the 60th % but less than or equal to the 80th %; greater than the 80th percentile score. These distinct cut-points were used to rank households into quintiles: poorest, poorer, middle, richer and richest, respectively. Table 1 presents the fixed format responses for each of these variables.

**Table 1:** Socio-Demographic Characteristics of Respondents (*N* = 14,779)

Variables	n (%),
**HIV/AIDS Status**	
Negative	1344 7 (91.0)
Positive	1332 (9.0)
**Gender**	
Male	7042 (47.6)
Female	7737 (52.4)
**Age**	
15–19	3406 (23.0)
20–24	2982 (20.2)
25–29	2207 (14.9)
30–34	2007 (13.6)
35–39	1720 (11.6)
40–44	1221 (8.3)
45–49	914 (6.2)
50+	322 (2.2)
**Education**	
No education	1341 (9.1)
Primary	8866 (60.0)
Secondary	4010 (27.1)
Post-Secondary	562 (3.8)
**Marital status**	
Never Married	4359 (29.5)
Currently Married	9176 (62.1)
Ever Married^a^	1244 (8.4)
**Religion**	
Catholic	2792 (18.9)
Church of Central Africa Presbyterian.	2589 (17.5)
Other Protestant Christians	7364 (49.8)
Islam	1767 (12.0)
No religion/others	266 (1.8)
**Age at first sex**	
Never had sex/never had a partner	1848 (12.5)
< 16	4609 (31.2)
16–17	3118 (21.1)
18–19	3008 (20.4)
20+	2196 (14.9)
**Total number of lifetime sexual partners**	
**0**	1848 (12.5)
1	4285 (29.0)
2	3423 (23.2)
3–4	3148 (21.3)
5–9	1427 (9.7)
10+	597 (4.0)
Undisclosed	51 (.3)
**Use of condom/use condom for most recent sex**	
No	9137 (61.8)
Yes	2155 (14.6)
Not had recent sex	3487 (23.6)
**Extra marital/partner sexual partners**	
None	12,128 (82.1)
1	2325 (15.7)
2+	325 (2.2)
Undisclosed	1 (0.0)
**Had any STI last 12 months**	
No	14,393 (97.4)
Yes	340 (2.3)
*Missing*	45 (.3)
**Household wealth index**	
Poor	5407 (36.6)
Rich	5799 (39.2)
Richest	3574 (24.2)
**Rural/urban Residence**	
Urban	2716 (18.4)
Rural	12,063 (81.6)
**Region**	
Northern	1809 (12.2)
Central	6368 (43.1)
Southern	6601 (44.7)

^a^Ever Married: included those who were divorced, separated and widowed

### Data collection

Data collection was done by trained enumeration officials from DHS. In all, a total of 32,040 individuals were included in the 2016 MDHS. A total of 24,562 women, aged 15–49 years, who were usually members of their households and those who spent the previous night in the selected households were interviewed. In a third of the selected households for the women’s survey, 7478 men, aged 15–54 years, who were regular members of their households or spent the previous night in the selected households were also interviewed. All men and women who were selected for the individual survey had HIV testing done after informed consent was obtained. After cleaning the data by dropping cases with missing or incomplete data on key variables and sample weight variable for HIV status, we included 14,779 cases for analysis. This may account for marginal discrepancies in the percentages as reported in the official DHS report. Demographic and anthropometric data were also obtained. Specific data collection themes that were relevant for the present study included age, sex, level of education, marital status, religion, age at first sex, total number of lifetime sexual partners, condom use for most recent sex, number of extramarital partner or sexual partners, previous STI in last 12 months, household wealth index, place and region of residence.

### Data access, preparation and analysis

The 2016 MDHS data used for analysis is freely available at www.dhsprogram.com and can be downloaded electronically after approval from the DHS program. De-identified data was downloaded from the DHS program website after permission was obtained by the first author, cleaned initially in SPSS and analyzed in STATA 16. Male and female data were downloaded separately and merged in SPSS. The initially merged file contained 32,040 cases in total, comprising 24,562 females and 7478 males. Likewise, HIV data was separately downloaded and merged with the combined male and female data that was initially merged to get the final data. Cases that had missing or incomplete data on HIV status and sample weight for HIV status were dropped. The eventual dataset for analysis consisted of 14,779 cases. All variables of interest were also identified. Univariate and bivariate analyses were weighted to account for sample weights in SPSS.

For the multivariate logistic regression estimates, a complex survey data analysis approach was adopted using the “svyset” command in STATA-16. This approach accounts for the complexities of sampling design employed in the DHS by adjusting for sampling clusters (*n* = 850), stratification (*n* = 56) and sample weights. This helps to prevent the potential bias of the standard errors associated with the confidence intervals (CI) of odds ratio estimates. Adjusted Odds Ratio (AOR) estimate and associated standard error and CI were reported in the multivariate results. We also performed regionally disaggregated analysis of HIV prevalence based on key demographic variables. A spatial map was also produced for visual presentation of HIV prevalence at the regional and district level using Quantum Geographical Information System (QGIS) [21] The district demarcation shapefile for Malawi was obtained from the free and open source GADM database [22].

### Spatial interpolation of HIV/AIDs epidemic at a scale lower than the region (clusters)

The second objective of this paper is to understand the HIV epidemic and inform programs and interventions at a lower geographical level. The 2016 Malawi DHS HIV testing data has information on HIV prevalence for 850 clusters. Each cluster had geolocation data (longitude and latitude), making it possible to determine the spatial variations of the HIV epidemic at the cluster level. We used the prevR package in the R freeware for statistical analysis [23]. This package was programmed to perform spatial estimation of regional trends of a prevalence using data from surveys with a stratified two-stage sample design [23]. Using functions available in the prevR package, we applied the gaussian kernel estimator approach with adaptive bandwidths of equal number of persons surveyed to generate a surface of HIV prevalence [23]. The main surface is a weighted estimate of HIV prevalence surface with parameter *N* = 368, a value chosen using the Noptim () function in the prevR package [23]. The ‘N’ is a function of the observed national prevalence, the number of persons tested and the number of clusters surveys, which are the three parameters used to simulate a DHS dataset [23]. The foreign package in R was used to read the data in R, and maptools and ggplot2 packages were used to display the Malawi HIV epidemic map. All these analyses were performed in R version 3.5.3 [24].

### Ethical considerations

Consent for enrolment into the DHS was obtained by enumeration officials on behalf of the National Statistical Office of Malawi and the DHS program. Consent for HIV testing was obtained from respondents and additional consent for the storage of blood samples for further testing was also sought. Consent for blood storage for further testing was independent of the consent for HIV testing. Therefore, one could opt-out of the storage of blood for further testing after consenting for the HIV test. In such cases, blood was discarded after confirmatory results for HIV test. Data obtained from the DHS is de-identified data before sharing with public, thus participants identifiers are removed, and no additional consents were obtained, and institutional ethical review was waived. One concern of using spatial data is the potential identification of respondents in their dwelling units on maps. However, this was addressed as the spatial data included only the coordinates of the center points of the clusters instead of the actual location of individual households. Moreover, the Global Positioning System (GPS) locations of the center points of the clusters were displaced at a random angle by up to 2 km and 5 km for urban and rural clusters, respectively. The DHS displacements of the GPS coordinates of the clusters is limited to the district boundaries of Malawi. Additionally, GPS locations for about 1% of the rural clusters were displaced by 10 km. Displacement was done before data was made available to the public. Although this helps to minimize the risk of identification of the households in the spatial analysis, it inherently makes the spatial analysis less accurate.

## Results

### Summary result of univariate analysis

A total of 14,779 participants were included in the final analysis. The result of the univariate analysis are presented in Table 1a slight majority were females (52.4%). The highest formal education for the majority was primary education (60%). The majority were currently married (62.1%) and were rural residents (81.6%). The prevalence of HIV was 9.0% among the participants. The details of the socio-demographic characteristic of respondents are presented in Table 1.

### Bivariate analysis results

Our bivariate analysis of the relationship between predictors and the prevalence of HIV infection is presented in Table 2. The relationship between HIV status and the independent variables were assessed using the chi-square test for independence. Except for the religious affiliation of respondents, all the independent variables were significantly associated with HIV status (Table 2). These included sex, age, educational status, marital status, age at first sex, recent sexual activity, total number of lifetime sexual partners, use condom for most recent sex, extramarital sexual partners, an STI in last 12 months, household wealth index, place of residence and region of residence.

**Table 2:** Results of Chi-square test for association between socio-demographic and HIV status (*N* = 14,779

Variables	HIV Status		Chi-square; *P*-value
	Negative	Positive	
	n (%)	n (%)	
**Gender**			χ2 = 60.894; *p* < 0.001
Male	6543 (92.9%)	499 (7.1%)	
Female	6904 (89.2%)	833 (10.8%)	
**Age**			χ2 = 647.472, *p* < 0.001
15–19	3333 (97.9%)	73 (2.1%)	
20–24	2862 (96.0%)	120 (4.0%)	
25–29	2031 (92.0%)	176 (8.0%)	
30–34	1742 (86.8%)	265 (13.2%)	
35–39	1461 (84.9%)	259 (15.1%)	
40–44	1013 (83.0%)	208 (17.0%)	
45–49	750 (82.0%)	165 (18.0%)	
50+	256 (79.5%)	66 (20.5%)	
**Education**			χ2 = 23.031, *p* < 0.001
No education	1177 (87.8%)	163 (12.2%)	
Primary	8092 (91.3%)	774 (8.7%)	
Secondary	3678 (91.7%)	332 (8.3%)	
Post-Secondary	499 (88.8%)	63 (11.2%)	
**Marital status**			χ2 = 448.486, *p* < 0.001
Never Married	4230 (97.0%)	129 (3.0%)	
Currently Married	8241 (89.8%)	934 (10.2%)	
Ever Married^a^	976 (78.5%)	268 (21.5%)	
**Religion**			χ2 = 6.103, *p* = 0.192
Catholic	2566 (91.9%)	227 (8.1%)	
Church of Central Africa Presbyterian.	2369 (91.5%)	220 (8.5%)	
Other Protestant Christians	6667 (90.5%)	697 (9.5%)	
Islam	1606 (90.9%)	161 (9.1%)	
No religion/others	238 (89.5%)	28 (10.5%)	
**Age at first sex**			χ2 = 139.621, *p* < 0.001
Never had sex/never had a partner	1815 (98.2%)	33 (1.8%)	
< 16	4111 (89.2%)	498 (10.8%)	
16–17	2821 (90.4%)	298 (9.6%)	
18–19	2719 (90.4%)	289 (9.6%)	
20+	1981 (90.3%)	214 (9.7%)	
**Recent Sexual activity**			χ2 = 142.181, *p* < 0.001
Never had sex	1815 (98.2%)	33 (1.8%)	
Active last 4 weeks	7618 (90.5%)	803 (9.5%)	
Not active last 4 weeks	4014 (89.0%)	496 (11.0%)	
**Total number of lifetime sexual partners**			χ2 = 390.753; *p* < 0.001
**0**	1815 (98.2%)	33 (1.8%)	
1	4081 (95.2%)	204 (4.8%)	
2	3045 (89.0%)	378 (11.0%)	
3–4	2710 (86.1%)	437 (13.9%)	
5–9	1262 (88.4%)	165 (11.6%)	
10+	490 (82.2%)	106 (17.8%)	
Undisclosed	43 (84.3%)	8 (15.7%)	
**Use condom for most recent sex**			χ2 = 27.044, *p* < 0.001
No	8269 (90.5%)	869 (9.5%)	
Yes	1931 (89.6%)	224 (10.4%)	
Not had recent sex	3247 (93.1%)	240 (6.9%)	
**Extra marital/partner sexual partners**			χ2 = 19.451, *p* < 0.001
None	11,008 (90.8%)	1120 (9.2%)	
1	2129 (91.6%)	196 (8.4%)	
2+	310 (95.4%)	15 (4.6%)	
Undisclosed	0 (0)	1 (100.0%)	
**Had any STI last 12 months**			χ2 = 62.122; *p* < 0.001
No	13,134 (91.2%)	1260 (8.8%)	
Yes	269 (79.1%)	71 (20.9%)	
*Don’t Know*	44 (97.8%)	1 (2.2%)	
**Household wealth index**			χ2 = 63.130; *p* < 0.001
Poor	5011 (92.7%)	396 (7.3%)	
Rich	5298 (91.4%)	500 (8.6%)	
Richest	3138 (87.8%)	436 (12.2%)	
**Rural/urban Residence**			χ2 = 144.740; *p* < 0.001
Urban	2309 (85.0%)	407 (15.0%)	
Rural	11,138 (92.3%)	925 (7.7%)	
**Region**			χ2 = 251.559; *p* < 0.001
Northern	1710 (94.5%)	100 (5.5%)	
Central	6005 (94.3%)	363 (5.7%)	
Southern	5732 (86.8%)	870 (13.2%)	

^a^Ever Married: included those who were divorced, separated and widowed

### Multivariate analysis results

The strength of association between independent variables and HIV status was assessed using multivariate complex samples logistic regression analysis. After adjusting for all other variables included in the multivariate analysis, independent predictors of the increased odds of HIV infection with reference to the base categories were: being female, in the higher age groups i.e. 25+ years, previous marital status, more than two total lifetime sexual partners, individuals who did not disclose status of extramarital partners, urban residency and residency in the southern region (Table 3). Factors associated with reduced odds of HIV infection (protective factors) were non-use of condom in most recent sexual activity, increasing number of extramarital partners, negative history of STI in the last 12 months, and being from a poor household. Unexpected findings were those of condom use and extramarital partners (Table 3). McKelvey and Zavoina’s R^2^ value for the model was 0.324, implying that the overall model explained 32.4% of the variability in HIV prevalence. Details of adjusted odds ratio (AOR) estimates and corresponding confidence intervals at 0.05 alpha level are presented in Table 3.

**Table 3:** Multivariate complex survey logistic regression estimates of HIV prevalence (*N* = 14,779)

Demographic Characteristic	OR	Std. Err.	*P*-value	95% CI of OR	
				Lower	Upper
**Gender**					
Male	Ref.				
Female	2.36	0.24	< 0.001	1.94	2.87
**Age**					
15–19	Ref				
20–24	1.38	0.30	0.14	0.90	2.10
25–29	2.43	0.51	< 0.001	1.61	3.66
30–34	4.02	0.85	< 0.001	2.66	6.08
35–39	4.90	0.95	< 0.001	3.35	7.19
40–44	5.83	1.15	< 0.001	3.95	8.60
45–49	5.91	1.29	< 0.001	3.85	9.08
50+	9.42	2.55	< 0.001	5.54	16.01
**Education**					
Post-Secondary	Ref.				
No education	1.06	0.32	0.85	0.59	1.90
Primary	1.25	0.32	0.39	0.75	2.07
Secondary	1.01	0.26	0.96	0.61	1.68
**Marital Status**					
Never Married	Ref.				
Currently Married	1.32	0.30	0.23	0.84	2.07
Ever Married^a^	1.88	0.41	< 0.001	1.22	2.90
**Religion**					
Other Protestants	Ref				
Catholic	0.90	.10	0.35	0.71	1.13
Church of Central Africa Presbyterian.	1.03	0.11	0.80	0.83	1.27
Muslim	0.77	0.10	0.05	0.59	1.00
No Religion/Other	1.53	0.49	0.18	0.82	2.85
**Total number of lifetime sexual partners**					
1	Ref.				
0	0.96	0.31	0.90	0.51	1.80
2	2.17	.24	< 0.001	1.75	2.69
3–4	3.12	0.39	< 0.001	2.45	3.97
5–9	3.10	0.54	< 0.001	2.21	4.36
10+	5.32	1.06	< 0.001	3.59	7.87
Undisclosed	2.88	1.55	0.05	1.01	8.26
**Use of condom/use condom for most recent sex**					
Yes	Ref				
No	0.57	0.07	< 0.001	0.45	0.72
Never had recent Sex	0.88	0.14	0.43	0.64	1.21
**Number of extra marital sex partners**					
**None**	Ref				
1	0.87	0.13	0.35	0.64	1.17
2+	0.40	0.16	0.02	0.18	0.89
Undisclosed	16.39	18.59	0.01	1.77	151.94
**Had any STI last 12 months**					
Yes	Ref				
No	0.57	0.11	< 0.001	0.39	0.82
Don’t know	0.23	0.25	0.18	0.03	1.93
**Household Wealth**					
Richest	Ref				
Poor	0.72	0.10	0.02	0.54	0.95
Rich	0.82	0.12	0.17	0.62	1.09
**Place of residence**					
Rural	Ref				
Urban	2.16	0.35	< 0.001	1.57	2.97
**Region of Residence**					
Northern Region	Ref				
Central region	1.09	0.19	0.63	0.77	1.53
Southern region	2.66	0.40	< 0.001	1.98	3.57
**Model Details**					
Population size	14,779				
Number of observations	14,779				
Number of strata	56				
Number of Primary Sampling Uuits	850				
Design df	794				
F (35, 760)	23.92				
Prob > F	< 0.001				
McKelvey and Zavoina’s R^2^	0.324				

^a^Ever Married: included those who were divorced, separated and widowed

The odds of HIV-positive status were 2.36 times greater in females compared to males, after adjusting for other variables in the model (AOR = 2.36, 95% CI = 1.94–2.87). The odds of HIV-positive status were 2.43 times greater among the age group of 25–29 years as compared to the age group of 15–19 years (AOR = 2.43, 95% CI = 1.61–3.66). The magnitude of this association increased steadily in the same direction as the years of the age groups increased (Table 3). As compared to those who had never-married, those who were ever-married (divorced, separated and widowed) had an 88% increased risk of being HIV-positive (AOR = 1.88, 95% CI = 1.22–2.90).

The odds of HIV-positive status were 2.17 times greater in people who reported two total number of lifetime sexual partners compared to those with only one lifetime sexual partner (AOR = 2.17, 95% CI = 1.75–2.69). This trend was maintained with increasing magnitude as the number of total lifetime partners increased. Unexpectedly, the odds of HIV-positive status decreased by 43% among people who had not used a condom in their most recent sexual activity as compared to those who had (AOR = 0.57, 95% CI = 0.45–0.72). People who reported two or more extramarital sexual partners had 60% decreased odds of being HIV-positive compared to their counterparts with no extramarital partners (AOR = 0.40, 95% CI = 0.18–0.89). The odds of being HIV-positive among those who did not disclose the number of extramarital sex partners was 16.4 times greater compared to those who indicated that they had no extramarital partners (AOR = 16.39, 95% CI = 1.77–151.94).

Relative to persons who had a previous STI in the past 12 months, the odds of HIV-positive status decreased by 43% among people with a negative history of STI in the last 12 months (AOR = 0.57, 95%CI = 0.39–0.82). The odds of HIV-positive status were 0.72 times lower in people from poorer households as compared to those from richest households (AOR = 0.72, 95% CI = 0.54–0.95). People residing in urban areas had a 2.2 times greater risk of being HIV-positive compared to their counterparts in the rural areas (AOR = 2.16, 95%CI = 1.57–2.97). Compared to those residing in northern Malawi, the odds of HIV prevalence were 2.66 times greater in southern Malawi (AOR = 2.66, 95% CI = 1.98–3.57).

### Regional distribution of HIV prevalence stratified by sociodemographic characteristics

Regional HIV prevalence in Malawi was disaggregated by gender. The disaggregated results of specific HIV prevalence with respect to the different socioeconomic and sexual risk behavior variables in each region are presented in the Supplementary Table 1.

**Fig. 1:**
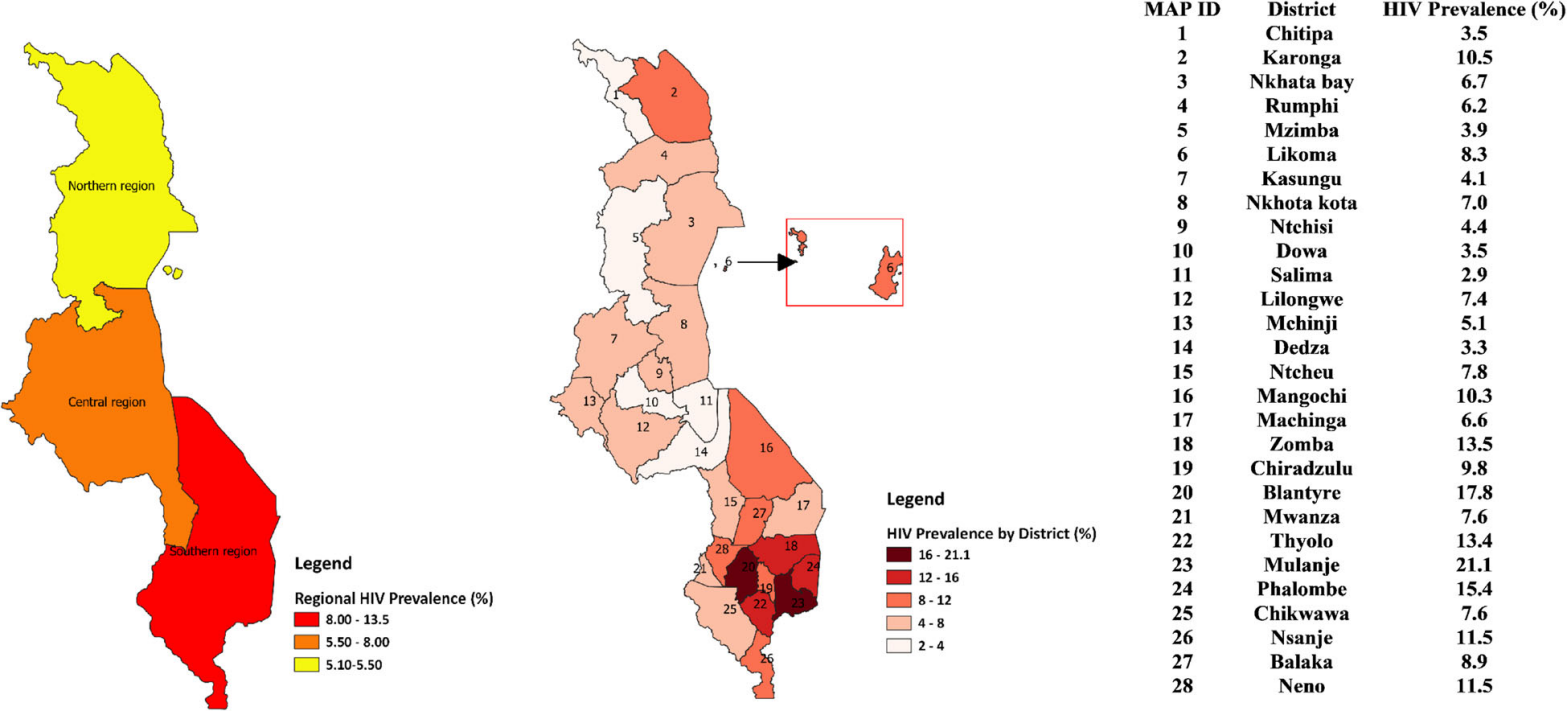
A map visualizing regional and district HIV prevalence in Malawi

Higher HIV prevalence was seen in the southern and central regions of Malawi (Fig. 1). HIV prevalence among females was consistently higher than their male counterparts in all three regions examined (Table S1). Uniformly across the three regions, HIV burden was higher in older age groups. Generally, the prevalence of HIV was highest among people with no education in northern and southern Malawi. In the central region, the prevalence was highest among those with post-secondary education (Table S1).

The burden of HIV was consistently higher among respondents who were ever-married in all the three regions. The prevalence HIV was 19.4% among people with no affiliated religion in the southern region (Table S1). Muslims in the Northern region had the highest burden (14.8%) relative to the central (7.1%) and southern (9.7%) regions. HIV prevalence by age of first sex showed that the southern region consistently had the highest burden irrespective of the age at first sex (Table S1).

Approximately, 12% of individuals in the southern region who reported one extramarital partner were HIV-positive, and 7.6% of individuals in the central region who reported at least two extramarital sex partners were HIV-positive (Table S1). Additionally, about 26.2 and 18.2% of individuals in the southern and northern regions, respectively, who reported a history of STI in the last 12 months were HIV-positive. With respect to household wealth, approximately 16.6% of the richest, 12.2% of rich and 11.9% of the poor in the south were HIV-positive (Table S1). Likewise, 19.2 and 11.9% of people in urban and rural areas in the southern region, respectively, were HIV-positive. In the central region, the HIV burden in urban and rural areas were 11.1 and 4.26%, respectively (Table S1). Generally, urban areas had a higher burden of HIV prevalence across the three regions (Table S1).

### HIV prevalence in Malawi estimated by kernel estimator approach

**Fig. 2:**
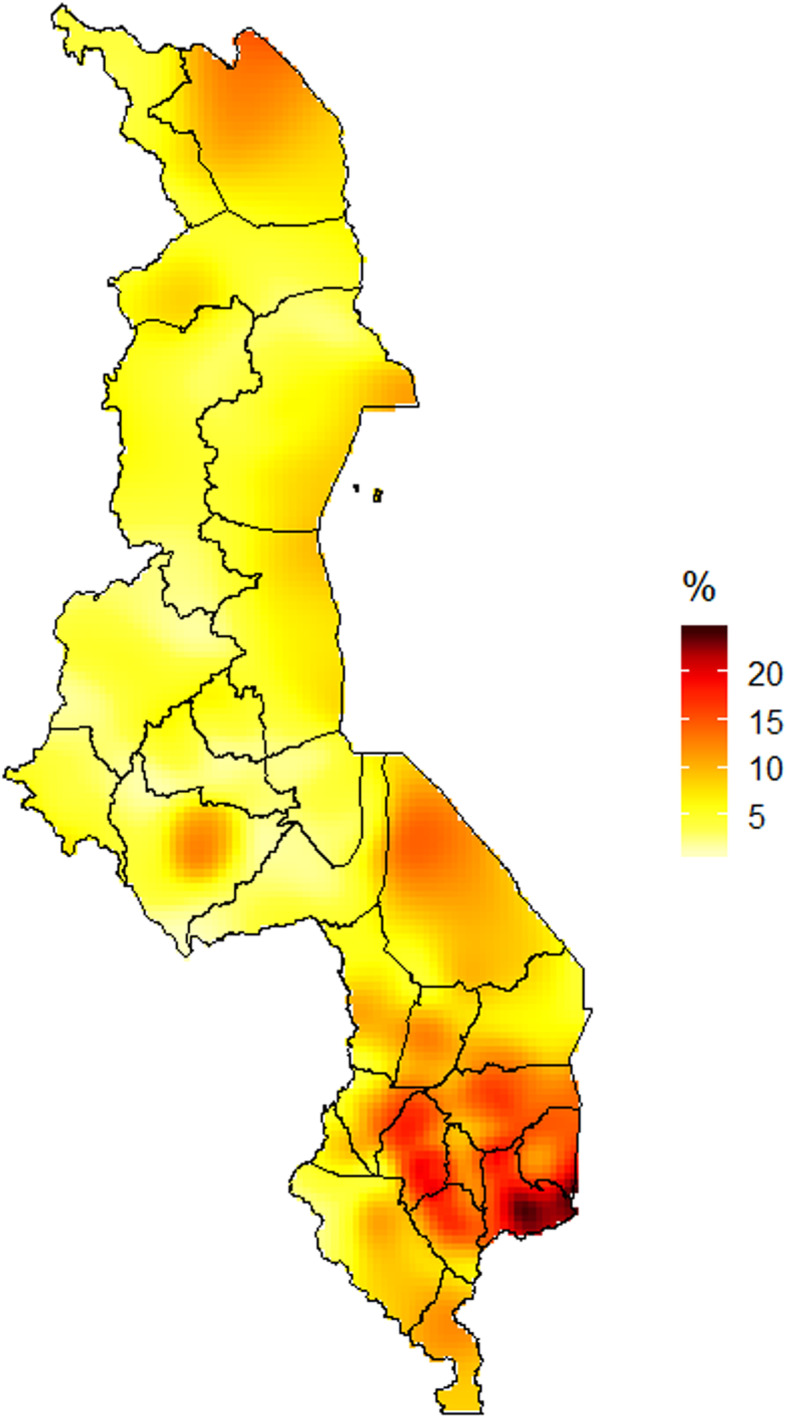
HIV prevalence in Malawi estimated by kernel estimator approach

We observed that the HIV epidemic is worse at the south-eastern part of Malawi (Fig. 1). The districts in the high prevalent zone of Malawi are: Thyolo, Zomba, Mulanje, Phalombe and Blantyre. In central and northern region, the district HIV prevalence map identified some zones that deserve attention (Fig. 1). The zone identified in the central region is Lilongwe (the national capital) and that of the northern region is Karonga (Fig. 1). Although the kernel estimator surface map emphasized the findings from the district HIV prevalence map, it revealed that there are variations in HIV prevalence within districts in Malawi (Fig. 2).

## Discussion

We examined the predictors of HIV infection in Malawi through a complex sample logistic regression and spatial mapping approach using the 2015–2016 Malawi Demographic and Health Survey (2016 MDHS) data. Even though similar studies were previously conducted in Malawi, to the best of our knowledge, this is the first study to use the 2016 MDHS dataset to build a complex samples logistic regression model of the predictors of HIV infection. In the overall analysis, gender, age, marital status, the total number of lifetime sexual partners, condom use and diagnosis with other STIs were all identified as significant predictors of HIV prevalence. Although these factors have previously been linked to HIV, their geographical-level distributions and contributions have not been well studied in Malawi.

We found that regional and sub-regional level variations exist in the prevalence of HIV in Malawi. Southern region has the highest HIV prevalence. The spatial analysis showed that the south-east region that covers Zomba, Mulanje, Blantyre, Phalombe, and Thyolo have high HIV prevalence rates. The high rates of HIV in the south-eastern region may result from a wide range of reasons that include cultural practices and low socioeconomic status. A study in Mulanje showed that risk of HIV transmission are attributed to the encouraging of girls to practice sex so they can be good wives [25, 26]. It is also important to note that this region boarders with Zambezia region of Mozambique. Zambézia region has an estimated HIV/AIDs prevalence of 15.1% [27]. The lack of rural health services especially in the Milange districts of Zambezia, Mozambique, pushes patients to access ART services in Malawi as it is closer [28], and this may also account for the high HIV prevalence in the region.

The high prevalence of HIV in the urban areas of the southern and central regions such as Lilongwe, Blantyre, Balaka and Zomba and in the transportation corridors such as Chipoka, Monkey Bay and Nsanje that connect to the neighboring countries such as Mozambique, Zimbabwe and Zambia, has been previously described [29, 30]. The southern and central region is home for the two major cities—Blantyre and Lilongwe, respectively—in addition to the old capital city Zomba, which together host the majority of the higher-income populations that are experiencing rapid urbanization. These regions also house the majority of the main tertiary institutions where many students, especially young women, are engaged in transactional sex and other risky sexual behaviors [31].

Our results are consistent with our understanding of HIV prevalence in Malawi. The high level of HIV prevalence among females compared to their male counterparts has been reported in previous studies [14, 32–34].

Regarding associated socioeconomic and behavioral factors of HIV, The results showed that HIV prevalence was high among females, those who were rich, were urban residents, were ever-married, had primary or no formal education and those with high-risk sexual behaviors. These findings are consistent with the results from previous studies based on the DHS data [8, 11, 16, 29, 35, 36]. It is important to note that by region, the majority of HIV-positive women live in the southern and central regions [37]. The low socio-economic status of women in the country that increases the engagement in transactional and commercial sex among young women, coupled with traditions such as polygamy and sexual cleansing ceremonies for young girls and widows, may explain the disproportionate prevalence of HIV among the female gender [26, 38]. However, another study also showed that women in Malawi are more at risk if they live in urban areas, have higher education, come from households with more wealth and were the heads of their households [37].

Furthermore, other reasons for the high rate of HIV infection among females could be due to widow inheritance and polygamy, which are common practices in some parts of southern and eastern Africa [39]. As a tradition in some African cultures, a woman whose husband is dead is forced to marry the husband’s younger brother to continue as a member of the family [40]. This is a common practice among two tribes (i.e. Nsanje and Mzimba) in Malawi, Kenya [39] and other parts of Africa [41]. In many cases, the younger brother may have multiple wives and other sexual partners; therefore, if the widow is HIV-positive from the previous marriage, her new husband is likely to get infected and also spread it to other wives and sexual partners [40].

A possible explanation for the high rate of HIV infections among ever-married individuals (i.e. those who were divorced, separated and widowed) could be that individuals who were previously married tend to have more sexual partners than single or married individuals [16]. Emina and colleagues found that HIV risk factors for Malawian women who were formerly in a union increased if the women had higher incomes or were the heads of their households, while for those who were in a union or never-married, the major predictors were living in urban areas and their age [37]. We assume that the pressure to provide for their families in an already hard economy may push some of these women into transactional sex, without disregarding that some of them may have been infected by their former partner before their divorce. Furthermore, the awareness of a partner’s HIV status and unsafe sexual behaviors could contribute to marriage breakdown, resulting in the association observed in our findings. Programs and interventions focusing on control of HIV/AIDS should focus on widowed, divorced and separated individuals as well as promoting appropriate prevention strategies such as condom use, use of post/pre-exposure prophylaxis and abstinence from sexual activities to prevent contracting HIV or other sexually transmitted infections (STIs).

Our results also confirmed a significant positive association between the number of lifetime sexual partners and increased chances of HIV infection. Lifetime sexual partners reported may be a proxy for sexual behavior and lifetime sexual history. A possible explanation for this finding is that an individual’s sexual behavior may change as a result of their HIV infection. Since awareness of HIV status is low in Malawi [42], changes in one’s lifetime sexual partner and for that matter, sexual behavior, are more likely to be due to an HIV-related illness or due to the loss of a regular partner due to HIV/AIDS.

While our study found a significant association between condom use for most recent sex and the prevalent of HIV infection, the percentage of individuals who tested positive for HIV and reported using a condom during the most recent sex (10.4%) was more than those who did not use condom a during the most recent sex (9.5%). There are two possible explanations to this unexpected finding: it is possible that most of the participants were aware of their HIV status and therefore were practicing safer sex to prevent HIV superinfection or infecting their partners, or given that the answer to the question was self-reported, there is a chance of recall and reporting bias.

Our results confirmed that spatial analyses of HIV prevalence are crucial for national AIDS programs when designing the most effective prevention strategies. To reduce new HIV infections, it is important to understand ‘where’ and ‘which populations’ should receive extra attention in terms of primary and secondary prevention activities such as HIV testing services, availability and accessibility of condoms, HIV education, initiating early antiretroviral treatment, formation and linkage to peer support groups and provision of pre- and post-exposure prophylaxis to prevent new HIV infections as well reduce HIV related mortality in Malawi. For instance, in Malawi, our results suggest that HIV prevention activities should be especially focused in the Zomba, Mulanje, Phalombe, Blantyre, and Thyolo districts in the southern, Lilongwe in the central region, and Karonga in the northern region. With regards to social, economic and behavioral characteristics, more attention should be paid to groups with higher prevalence of HIV such as women, those who are widowed, divorced or separated, the richest, people older than 25 years, those engaged in extramarital affairs, urban dwellers, those who have no formal education, and those with STIs. An intervention that should be considered in the southern region is to discourage the cultural practice of sex initiation that seek to prepare young girls as good wives.

Our study has some strength and limitations that deserve highlighting. A major strength of our study was the use of large, nationally representative survey data set (2016 MDHS) which was based on a standardized methodology for analyses. Secondly, the study employed a complex sample analytic design to account for sampling units, stratification and weighting. The study also employed spatial analytical techniques that has advantages over standard statistical techniques to identify geographical variations of HIV prevalence in Malawi. These spatial maps help to visualize HIV prevalence at the sub-district, district and regional levels. Our findings, however, are subject to limitations that must be taken into consideration. One limitation of using spatial maps to visualize HIV burden is that the prevalence is dispersed across all pixels even though some areas have no population. Moreover, as a characteristic of all cross-sectional studies, this study could neither establish temporality nor causality of the observed associations of the predictors with the prevalence of HIV infection. Secondly, self-reporting of sexual behaviors is prone to recall and reporting bias. Despite these limitations, this study of population-level spatial analysis of HIV prevalence in Malawi.

## Conclusion

Our results emphasize the importance of geographical-level variations and the impact of well-established factors including sociodemographic, sexual behaviors and biological factors for HIV prevalence. The districts in the high HIV prevalent zone of Malawi are Thyolo, Zomba, Mulanje, Phalombe and Blantyre. In central and northern region, the district HIV prevalence map identified Lilongwe in the central region and Karonga in the northern region as districts that equally deserve attention. The findings of the study have shown that factors such as: female gender, age above 25 years, place of residence, widowed, divorced and separated marital status, increased number of lifetime sexual partners, extramarital sexual activity and diagnosis with other STIs are important predictors of HIV infection in Malawi. This study could help Malawian public health officials to develop HIV prevention programs by showing where and which populations need more resources and attention. Our findings might also encourage health policymakers in other resource-limited countries to apply spatial analysis or other regionally segregated analysis to identify areas they need to target for designing an intervention to reduce the spread of HIV and guide the treatment and management of HIV related illnesses.

## Supplementary information

**Additional file 1: Table S1.** Summary Statistics of Study Variables (*N* = 14,779).

## Data Availability

The datasets analyzed during the current study are publicly available and can be obtained upon a simple, registration-access request at the following DHS web address https://dhsprogram.com/data/dataset_admin/index.cfm.

## Abbreviation

AIDS: Acquired Immune Deficiency Syndrome
AOR: Adjusted Odds Ratio
AIS: AIDS Indicator Survey
CI: Confidence Interval
DHS: Demographic Health Survey
GPS: Global Positioning System
HIV: Human Immunodeficiency Virus
MDHS: Malawi Demographic Health Survey
QGIS: Quantum Global Positioning System
STI: Sexually Transmitted Infection
WHO: World Health Organization
