# Supplementary material for: Spatial analysis of factors associated with HIV infection in Malawi: indicators for effective prevention
Source: BMC Public Health. 2020 Jul 25;20:1167. doi: 10.1186/s12889-020-09278-0 (PMC7382788; doi:10.1186/s12889-020-09278-0)
Supplement: Supplementary file 1 — Additional file 1: Table S1. Summary Statistics of Study Variables (N = 14,779). [file 12889_2020_9278_MOESM1_ESM.docx]

Table 1: Summary Statistics of Study Variables (N=14779)

| **Region Name** | **Northern region** | **Central region** | **Southern region** |
| --- | --- | --- | --- |
|  | Weighted Prevalence (%) | Weighted Prevalence (%) | Weighted Prevalence (%) |
| **Age Groups (yrs)** |  |  |  |
| 15-19 | 0.5 | 1.8 | 2.9 |
| 20-24 | 2.1 | 2.9 | 5.6 |
| 25-29 | 4.3 | 4.6 | 12.7 |
| 30-34 | 7.9 | 8.5 | 19.3 |
| 35-39 | 9 | 10.3 | 21.2 |
| 40-44 | 11.5 | 10.3 | 24.8 |
| 45-49 | 11.2 | 10.1 | 27 |
| 50+ | 25.6 | 9 | 29.5 |
| **Gender** |  |  |  |
| Male | 5.4 | 4.6 | 10.2 |
| Female | 5.6 | 6.8 | 15.7 |
| **Education** |  |  |  |
| No Education | 14.9 | 7.3 | 15.6 |
| Primary | 5.5 | 4.7 | 13.6 |
| Secondary | 5 | 6.5 | 11 |
| Higher Education | 4.3 | 10.5 | 15.6 |
| **Marital Status** |  |  |  |
| Never Married | 1.5 | 2.3 | 4 |
| Currently Married | 6.3 | 6.3 | 15.2 |
| Ever Married | 14.6 | 13.8 | 29.4 |
| **Religion** |  |  |  |
| Catholic | 6.1 | 5 | 12.5 |
| Church of Central Africa Presbyterian | 4.5 | 5.4 | 17.2 |
| Protestant Christians | 5.4 | 5.9 | 13.8 |
| Islam | 14.8 | 7.1 | 9.7 |
| No Religion/Others | 0 | 6.8 | 19.4 |
| **Age at First Sex** |  |  |  |
| Never Had Sex | 0 | 1.2 | 3 |
| Under Age16 | 6 | 7.3 | 14.3 |
| 16-17 | 6.2 | 6.2 | 13.5 |
| 18-19 | 6 | 6.5 | 14.5 |
| 20Above | 8.2 | 4.8 | 16.9 |
| **Recent Sex Activity** |  |  |  |
| Never had sex | 0 | 1.2 | 3 |
| Active in Last 4weeks | 6.3 | 5.7 | 14.5 |
| Not Active in Last Weeks | 6.6 | 7.8 | 14.6 |
| **Total lifetime sex partners** |  |  |  |
| 0 | 0 | 1.2 | 3 |
| 1 | 2.7 | 3.7 | 6.8 |
| 2 | 7 | 6.1 | 16.4 |
| 3 - 4 | 7.8 | 9.5 | 18.8 |
| 5 - 9 | 13 | 5.9 | 16.1 |
| 10 and above | 12.3 | 14 | 22 |
| Undisclosed | 25 | 6.7 | 17.9 |
| **Condom Use in Most Recent Sex** |  |  |  |
| No Condom | 5.9 | 6 | 13.9 |
| Yes Condom | 7.5 | 5.4 | 15.2 |
| No Recent Sex | 3.2 | 5.1 | 9.8 |
| **Extra Marital Sex** |  |  |  |
| None | 5.2 | 5.9 | 13.8 |
| Extra1 | 6.9 | 4.5 | 11.9 |
| Extra 2+ | 5.3 | 7.6 | 2.9 |
| **STI Diagnosis in Last 12 Months** |  |  |  |
| No | 5.3 | 5.5 | 12.9 |
| Yes | 18.2 | 14.8 | 26.2 |
| Don't Know | 0 | 0 | 4 |
| **Wealth Index** |  |  |  |
| Poor | 6 | 3.6 | 11.9 |
| Rich | 5.7 | 5.3 | 12.2 |
| Richest | 5 | 10.2 | 16.6 |
| **Place of Residence** |  |  |  |
| Urban | 6.4 | 13.4 | 19.2 |
| Rural | 5.3 | 3.9 | 11.9 |
